# Artificial intelligence in mental healthcare: transformative potential vs. the necessity of human interaction

**DOI:** 10.3389/fpsyg.2024.1378904

**Published:** 2024-12-17

**Authors:** Anithamol Babu, Akhil P. Joseph

**Affiliations:** ^1^School of Social Work, Marian College Kuttikkanam Autonomous, Kuttikkanam, India; ^2^School of Social Work, Tata Insititute of Social Sciences Guwahati-Off Campus, Jalukbari, India

**Keywords:** artificial intelligence, mental healthcare, personalization, ethics in AI, accessibility in healthcare

The integration of artificial intelligence (AI) into mental healthcare represents a profound shift, merging cutting-edge technology with the intricate and deeply personal dynamics of human psychology. While AI's potential to revolutionize diagnosis, treatment, and access to mental healthcare is undeniable, it has sparked a critical debate. Can AI truly replicate the essential human touch foundational to therapeutic success, or does it enhance mental healthcare by addressing inefficiencies such as limited access, personalization, and diagnostic precision? This article argues that AI's potential in mental healthcare is transformative, complementing rather than replacing human therapists, and will outline major arguments in personalization, accessibility, ethics, and human-AI balance.

## Enhancing personalization and diagnostic precision with AI

Traditional therapeutic models, while human-centered, often rely on generalizations due to limited time, resources, and the subjective nature of the diagnosis (Stein et al., 2022). Mental health conditions like depression, anxiety, and schizophrenia are typically diagnosed based on symptoms, leading to generalized treatments that often overlook individual patient needs (Wakefield, 2007). This approach risks limiting care to symptom management rather than personalized interventions. AI challenges this approach by offering unprecedented levels of personalization through data analysis (Johnson et al., 2021). AI systems can process vast data from various sources, such as speech patterns, behavioral analytics, physiological responses, and genetic information, providing a comprehensive understanding of a patient's mental health (Thakkar et al., 2024). Machine learning algorithms are capable of recognizing patterns that human therapists might overlook, offering insights into mood fluctuations, cognitive distortions, and even early signs of psychosis (Zhou et al., 2022). By analyzing data at this granular level, AI enables clinicians to tailor interventions specifically to an individual's psychological and biological makeup, thus offering highly personalized treatment plans. Recent studies have demonstrated that AI can predict patient responses to different therapeutic modalities, adjusting treatment strategies dynamically, far more accurately than traditional methods (Sezgin and McKay, 2024; Cho et al., 2020).

However, while AI offers promise for improving diagnostic precision, it has limitations, as its accuracy depends on the quality of the data it is trained on. Incomplete or biased datasets can lead to significant diagnostic errors, particularly in diverse populations, by misinterpreting symptoms or overlooking the complexity of mental health conditions. This risk is especially pronounced in mental health, where inappropriate treatments can severely impact patient wellbeing (Yan et al., 2022). For instance, a study in Ethiopia found that 39.16% of patients with severe psychiatric disorders were misdiagnosed, with rates higher among non-specialists (Ayano et al., 2021). Similarly, a Canadian study reported high misdiagnosis rates among 840 primary care patients: 65.9% for major depressive disorder, 92.7% for bipolar disorder, and over 70% for anxiety disorders (Vermani et al., 2011). Such findings underscore the inherent challenges in mental health diagnosis, which often relies on subjective doctor-patient interactions prone to inaccuracy (Yan et al., 2022). Moreover, a shortage of psychiatrists, particularly in developing countries, exacerbates the issue (Sholevar et al., 2017). In contrast, machine-based diagnoses offer several advantages, including conserving human resources, increasing efficiency, enabling large-scale assessments, and potentially reducing stigma (Ueda et al., 2024); however, over-reliance on AI without adequate human oversight risks perpetuating, rather than resolving, existing issues in mental healthcare. While AI enhances diagnostics through real-time data and predictive modeling, it must be complemented by the clinical judgment of experienced professionals, as it cannot fully capture the complexity of human emotions, behaviors, and cultural factors (Graham et al., 2019; Loscalzo et al., 2017; Khare et al., 2024). Clinicians must ensure AI remains a supportive tool, not a replacement, and address risks like biased data to safeguard patient care quality (Ueda et al., 2024).

## Bridging the accessibility gap: AI as a tool for mental health equity

The issue of accessibility in mental healthcare is a pressing concern, as many individuals, particularly in underserved or rural areas, struggle to access qualified mental health professionals (Morales et al., 2020). Despite the growing awareness of mental health issues, barriers such as high costs, long wait times, and overburdened healthcare systems make therapy inaccessible for a significant portion of the population (Kourgiantakis et al., 2023). This is where AI's role as a democratizing force becomes particularly relevant. AI-driven mental health platforms, like Woebot and Wysa, offer cost-effective alternatives to traditional therapy by providing digital interventions, particularly in cognitive-behavioral therapy (Haque and Rubya, 2023). These platforms can scale therapeutic support, delivering ongoing mental healthcare to individuals who may otherwise be left without any form of assistance due to financial constraints or geographic limitations, especially where human therapists are scarce (Fitzpatrick et al., 2017).

However, the belief that AI will automatically democratize mental healthcare is overly optimistic and overlooks substantial challenges. While AI platforms can offer scalable solutions, they fail to address systemic issues related to the digital divide. Many rural and low-income populations lack the technological infrastructure—such as reliable internet access, smart devices, and digital literacy—needed to benefit from AI-driven mental health interventions (Kozelka et al., 2023). Without addressing these foundational disparities, AI cannot effectively bridge the mental healthcare gap and may, instead, deepen existing inequalities. Governments and healthcare providers must invest in AI platforms and in building the necessary infrastructure and providing digital education to ensure that the most vulnerable populations can engage with these tools. According to the World Health Organization, AI's potential to reduce disparities in mental healthcare can only be realized if these systemic barriers are addressed alongside the deployment of AI-driven solutions (Khan et al., 2023).

## Precision without compromise: AI's role in predictive mental healthcare

One of the significant advantages of AI in mental healthcare is its capacity for real-time monitoring and predictive analytics, particularly in managing chronic conditions like mood disorders, and schizophrenia (Thakkar et al., 2024). AI systems can continuously track patients' behavior, mood, and cognitive patterns, identifying early warning signs of relapse or deterioration before they become noticeable to clinicians (Cho et al., 2020). This enables early intervention, which can be crucial in preventing severe crises such as suicide attempts or hospitalizations. A study by Lee et al. (2021) found that AI systems could predict mood fluctuations and relapse risk in patients with mood disorders more accurately than human clinicians. By analyzing behavioral data and patient history, AI systems can foresee when a depressive episode is likely, allowing for tailored treatments or medication adjustments that can potentially alter the course of a patient's recovery.

However, while these capabilities offer clear benefits, the psychological impact of continuous AI monitoring raises significant concerns that are often overlooked. Constant surveillance could lead to feelings of anxiety, hypervigilance, or even a loss of privacy, as patients might feel reduced to data points rather than being treated as individuals with complex emotional experiences (Joseph and Babu, 2024). This can affect the therapeutic alliance between the patient and clinician, central to effective care. If patients perceive that their every behavior is being monitored by machines, the human connection fundamental to therapy may erode, creating a sense of detachment or mistrust (Prasko et al., 2022). The ethical implications of AI-driven monitoring must be critically examined, particularly regarding how it may shift the power dynamic in therapy, with patients feeling scrutinized by technology rather than supported by a human therapist. Maintaining human oversight and ensuring that AI supports, rather than undermines, the therapeutic relationship is essential (Alowais et al., 2023).

## Ethical and privacy challenges in AI-driven mental healthcare

Integrating AI into mental healthcare raises serious ethical concerns, particularly regarding data privacy and algorithmic bias, both of which pose significant challenges beyond the technological safeguards currently in place (Warrier et al., 2023). Mental health data is among the most sensitive types of personal information, and the risk of misuse or data breaches can have devastating consequences for patients, including stigmatization, loss of employment, or insurance discrimination (Seh et al., 2020). Despite strong privacy protections like encryption and anonymization, real-world cases such as the Vastaamo data breach in Finland, where 36,000 psychotherapy records were compromised, highlight the vulnerability of AI systems to exploitation (Ghanbari and Koskinen, 2024; Inkster et al., 2023). As Gentili (2021) highlights, technological interventions in complex systems like the human mind create “Bio-ethical Complexity,” “Bio-ethical Complexity” raises concerns about relying solely on AI in mental healthcare, especially as cyberattack risks grow. While robust privacy measures must be continuously updated, human error and system flaws remain significant challenges.

Algorithmic bias in AI systems necessitates thorough examination, as AI models reflect the biases present in their training data, often mirroring societal inequalities across race, gender, socioeconomic status, and culture. This bias can lead to skewed diagnoses and treatment recommendations, exacerbating healthcare disparities rather than alleviating them (Celi et al., 2022). Recent cases highlight AI bias in healthcare: a U.S. hospital algorithm assigned lower risk scores to Black patients than to white patients with similar health conditions, limiting their access to care (Ledford, 2019). Another case showed a skin cancer detection model misdiagnosing darker skin tones due to predominantly white training data, reducing accuracy for non-white patients (Krakowski et al., 2024). Such examples underscore that merely refining algorithm or incorporating diverse datasets is insufficient; systemic changes in data collection, interpretation, and application are required to capture a more comprehensive and equitable view of patient needs. AI's feedback loops can entrench biases, making them harder to eliminate over time (Ferrara, 2024). Addressing this requires not just diverse datasets but also identifying implicit biases and maintaining rigorous oversight. Without these measures, AI risks deepening, rather than reducing, healthcare disparities.

## A collaborative approach: merging AI and human expertise in therapy

AI's ability to analyze extensive datasets and detect patterns that may escape human therapists offers a significant advantage, particularly in areas such as diagnostic precision and individualized care. However, AI lacks the emotional intelligence and cultural sensitivity intrinsic to human therapists, whose expertise extends beyond data to include empathy, intuition, and non-verbal communication—all critical for effective mental healthcare (Minerva and Giubilini, 2023). Excessive reliance on AI risks overshadowing the therapist's clinical judgment and intuition, potentially reducing therapy to a mechanistic process devoid of human warmth and understanding (Prasko et al., 2022). Over-dependence on AI can lead to “automation bias,” where clinicians place excessive trust in machine recommendations, which may erode their role as primary decision-makers and affect the quality of personalized care. While patient perspectives on AI are mixed—some appreciate its accessibility, while others feel it may compromise the human connection in therapy—these concerns underscore the importance of implementing patient-centered AI tools that supplement, rather than replace, therapist-patient interactions (Ali et al., 2023; Sathyan et al., 2022). Explainable AI (XAI) tools, such as SHAP (Shapley Additive exPlanations) and LIME (Local Interpretable Model-Agnostic Explanations), address these challenges by providing transparency in AI decision-making, allowing therapists to understand and validate AI insights without fully relinquishing control, while continuous professional development ensures that therapists use AI as a supportive tool rather than allowing it to dominate their decisions (Ali et al., 2023; Minerva and Giubilini, 2023). The future of AI-augmented therapy hinges on maintaining a balance between AI's precision and the therapist's empathy, fostering a collaborative model that enhances rather than diminishes the core relational elements of mental healthcare (Table 1).

**Table 1:** Collaborative integration of AI and human therapists in mental healthcare.

**Aspect**	**AI's role**	**Human therapist's role**	**Collaborative outcome**
Data analysis	Processes large datasets to recognize behavioral patterns	Interprets data insights with personal patient knowledge	Combines AI insights with therapist understanding for a more comprehensive view of the patient's needs
Real-time monitoring	Tracks mood fluctuations and behavioral changes continuously	Responds empathetically to real-time patient needs	Supports timely interventions, allowing the therapist to address issues as they arise
Pattern recognition	Identifies patterns that may be imperceptible to humans	Applies nuanced, contextual understanding	Detects subtle indicators while incorporating patient history and context
Empathy and connection	Lacks emotional intelligence	Provides empathetic, individualized care	Ensures that AI's efficiency is complemented by genuine human empathy, maintaining patient trust
AI-assisted diagnosis	Offers precision in symptom analysis and diagnostic support	Confirms diagnosis with holistic patient assessment	Reduces diagnostic errors by merging AI precision with therapist's intuitive judgment
Patient trust	Enhances treatment accuracy but may seem impersonal	Builds a strong therapeutic alliance with patients	Combines efficient, accurate treatment plans with a personal, trusting relationship between therapist and patient
Ethical judgment	Operates within programmed constraints	Makes ethical decisions based on context	Aligns AI's objective analysis with human ethical judgment to provide safe and fair patient care

## AI as a complementary tool in mental healthcare

AI is a transformative force in mental healthcare, but its integration must balance technological precision with human empathy, ensuring that it complements, rather than replaces, the essential therapeutic relationship. As Topol (2019) notes, the convergence of AI and human intelligence has the potential to revolutionize healthcare by harnessing both systems' strengths. Complexity Science suggests a holistic approach, integrating dimensions like ethical, philosophical, religious, cultural and emotional dimensions with technological innovations, ensuring empathy and precision coexist in mental health treatment. Addressing adoption complexities—such as regulation, scalability, cost, and practitioner acceptance—requires robust infrastructure, phased implementations, pilot programs, and AI-human collaboration models to ensure safety, privacy, and equitable access like Wysa's approach to privacy concerns and adaptability across languages and cultures (Dinesh et al., 2024). Long-term sustainability also demands updates, ethical oversight, and resources to prevent biases and inconsistent care. While AI benefits early intervention, it may affect the therapeutic alliance, with continuous monitoring risking feelings of surveillance. Thus, AI should remain a complementary tool, carefully integrated to preserve the emotional and relational elements essential to mental healthcare. This article calls for actionable steps—such as ethical AI investment and patient-centered design—to bridge human-AI gaps in mental healthcare.
